# Vaccine effectiveness against SARS-CoV-2 reinfection during periods of Alpha, Delta, or Omicron dominance: A Danish nationwide study

**DOI:** 10.1371/journal.pmed.1004037

**Published:** 2022-11-22

**Authors:** Katrine Finderup Nielsen, Ida Rask Moustsen-Helms, Astrid Blicher Schelde, Mie Agermose Gram, Hanne-Dorthe Emborg, Jens Nielsen, Christian Holm Hansen, Michael Asger Andersen, Marianna Meaidi, Jan Wohlfahrt, Palle Valentiner-Branth

**Affiliations:** 1 Department of Infectious Disease Epidemiology and Prevention, Statens Serum Institut, Copenhagen, Denmark; 2 Department of Data Integration and Analysis, Statens Serum Institut, Copenhagen, Denmark; 3 Department of Epidemiology Research, Statens Serum Institut, Copenhagen, Denmark; Burnet Institute, AUSTRALIA

## Abstract

**Background:**

Individuals with a prior Severe Acute Respiratory Syndrome Coronavirus 2 (SARS-CoV-2) infection have a moderate to high degree of protection against reinfection, though seemingly less so when the Omicron variant of SARS-CoV-2 started to circulate. The aim of this study was to evaluate the vaccine effectiveness (VE) against SARS-CoV-2 reinfection, Coronavirus Disease 2019 (COVID-19)-related hospitalization, and COVID-19-related death, in individuals with prior SARS-CoV-2 infection, and to assess the effect of time since vaccination during periods with different dominant SARS-CoV-2 variants.

**Methods and findings:**

This study used a nationwide cohort design including all individuals with a confirmed SARS-CoV-2 infection, who were alive, and residing in Denmark between 1 January 2020 and 31 January 2022. Using Danish nationwide registries, we obtained information on SARS-CoV-2 infections, COVID-19 vaccination, age, sex, comorbidity, staying at hospital, and country of origin. The study population included were individuals with prior SARS-CoV-2 infection. Estimates of VE against SARS-CoV-2 reinfection with 95% confidence intervals (CIs) were calculated using a Poisson regression model and adjusted for age, sex, country of origin, comorbidity, staying at hospital, calendar time, and test incidence using a Cox regression model. The VE estimates were calculated separately for three periods with different dominant SARS-CoV-2 variants (Alpha (B.1.1.7), Delta (B.1.617.2), or Omicron (B.1.1.529)) and by time since vaccination using unvaccinated as the reference. In total, 148,527 person-years and 44,192 SARS-CoV-2 infections were included for the analysis regarding reinfections. The study population comprised of 209,814 individuals infected before or during the Alpha period, 292,978 before or during the Delta period, and 245,530 before or during the Omicron period. Of these, 40,281 individuals had completed their primary vaccination series during the Alpha period (19.2%), 190,026 during the Delta period (64.9%), and 158,563 during the Omicron period (64.6%). VE against reinfection following any COVID-19 vaccine type administered in Denmark, peaked at 71% (95% CI: -Inf to 100%) at 104 days or more after vaccination during the Alpha period, 94% (95% CI: 92% to 96%) 14 to 43 days after vaccination during the Delta period, and 60% (95% CI: 58% to 62%) 14 to 43 days after vaccination during the Omicron period. Waning immunity following vaccination was observed and was most pronounced during the Omicron period. Due to too few events, it was not possible to estimate VE for hospitalization and death. Study limitations include potentially undetected reinfections, differences in health-seeking behavior, or risk behavior between the compared groups.

**Conclusions:**

This study shows that in previously infected individuals, completing a primary vaccination series was associated with a significant protection against SARS-CoV-2 reinfection compared with no vaccination. Even though vaccination seems to protect to a lesser degree against reinfection with the Omicron variant, these findings are of public health relevance as they show that previously infected individuals still benefit from COVID-19 vaccination in all three variant periods.

## Abstract 2

Katrine Finderup Nielsen and colleagues investigate vaccine effectiveness against SARS-CoV-2 reinfection, COVID-19 related hospitalization and COVID-19 related death, in previously infected individuals in Denmark.

## Author summary

**Why was this study done?:**

- Vaccination is one of the best tools we have to curb the spread of Coronavirus Disease 2019 (COVID-19).
- This study was conducted to gain knowledge on whether previously infected individuals would still benefit from vaccination against COVID-19.

**What did the researchers do and find?:**

- Using nationwide, Danish register data for three separate periods of different Severe Acute Respiratory Syndrome Coronavirus 2 (SARS-CoV-2) variant dominance (Alpha, Delta, Omicron), more than 200,000 previously infected individuals were included in each period.
- For each period, the vaccine effectiveness (VE) against SARS-CoV-2 reinfection, COVID-19-related admission, and COVID-19-related death was investigated.
- This study showed that among previously infected individuals who have completed a primary vaccination series, vaccines are still effective against SARS-CoV-2 reinfection during periods with SARS-CoV-2 variants Alpha, Delta, and Omicron, ranging from 60% (95% confidence interval (CI): 58% to 62%) to 94% (95% CI: 92% to 96%) and lasting up to 9 months.

**What do these findings mean?:**

- These findings show that previously infected individuals still benefit from COVID-19 vaccination during all three variant periods.
- Insight into VE in individuals with natural immunity is important to help decision makers plan vaccination strategies.
- Unmeasured biases such as changes in risk behavior might influence the result, but the completeness of the national, Danish registries counteract this.
- Future studies with longer follow-up time are necessary to ascertain VE against severe outcomes in those with previous infection.

## Introduction

Previous observational studies have investigated the association between Coronavirus Disease 2019 (COVID-19) vaccination and Severe Acute Respiratory Syndrome Coronavirus 2 (SARS-CoV-2) reinfection [1–3], but the duration and effect of protection from vaccination after a SARS-CoV-2 infection remains of public health interest. Studies show that natural immunity is more potent in protecting against SARS-CoV-2 infections than vaccination is in SARS-CoV-2 naïve individuals [4,5], and waning is seen for both types of immunity but less so for the naturally induced [5]. Despite an estimated moderate to high natural protection against reinfection with non-Omicron variants of SARS-CoV-2 [6–8], data from Denmark and Qatar suggest a lower protection against reinfection with the Omicron (B.1.1.529) variant [7,8]. Therefore, it is of great public health concern to examine the additional benefits of vaccination among individuals with a history of SARS-CoV-2 infection.

In Denmark, the healthcare system provides universal healthcare to everyone residing in Denmark [9], guaranteeing access to free COVID-19 testing and vaccines as well as medical care. A COVID-19 vaccination program was rolled out in increments from end of December 2020, prioritizing those with increased exposure to SARS-CoV-2 or risk of severe COVID-19 [10]. A booster vaccination campaign was rolled out in the same manner from September 2021. Vaccines administered in Denmark were Comirnaty (BNT162b2), Spikevax (mRNA-1273), Vaxzevria (ChAdOx1), and Jcovden (Ad26.COV2-S).

The study objective was to examine vaccine effectiveness (VE) against SARS-CoV-2 reinfection, COVID-19-related hospitalization, and COVID-19-related death, in previously infected individuals, and to assess the effect of time since vaccination (waning of immunity following vaccination) in calendar periods where the SARS-CoV-2 variants Alpha (B.1.1.7), Delta (B.1.617.2), or Omicron (B.1.1.529) were dominant.

## Methods

### Data extraction and preparation

The Danish Civil Registration System (CRS) holds information on date of birth, emigration, immigration, and death of all individuals in Denmark [11]. The CRS also holds a unique personal registration number for all residents in Denmark. Information on SARS-CoV-2 infections, defined as a positive SARS-CoV-2 reverse transcription polymerase chain reaction (RT-PCR) test, was obtained from the Danish Microbiology Database (MiBa), which is a national database containing real-time information on all microbiological laboratory test results from all clinical microbiology and private test centers, including negative tests and date of sampling [12]. Information on all COVID-19 vaccines (exposure) was obtained from the Danish Vaccination Registry (DVR). All vaccinators are obliged to document administered vaccines in this registry [13]. Information on comorbidities were retrieved from the Danish National Patient Registry (DNPR) [14]. A primary vaccination series was defined as two doses COVID-19 mRNA vaccine (Comirnaty or Spikevax), two doses Vaxzevria, or one dose Jcovden. In Denmark, the use of Vaxzevria was halted, and those who had received one dose Vaxzevria was subsequently offered one dose mRNA vaccine. This mixed regimen was registered as an mRNA primary vaccination series since it has been shown that mixed vaccination yields a VE comparable to that of two mRNA vaccines [15]. A mix of mRNA vaccines was also considered a primary vaccination series and registered by the brand of the second dose. By using the unique personal registration number and combining information from the CRS, MiBa, DVR, and DNPR, we identified all individuals who were alive and residing in Denmark between 1 January 2020 and 31 January 2022, and who had a confirmed SARS-CoV-2 infection during the study period. These individuals constitute the study population. Rapid antigen test results are also recorded in MiBa but were not included in the analyses due to low sensitivity [16]. Individuals with a positive antigen test were urged to confirm the result by RT-PCR. We applied a 90-day window following a laboratory confirmed RT-PCR SARS-CoV-2 positive test to avoid ongoing infections being misclassified as new infections. The following outcomes were investigated: SARS-CoV-2 reinfection, COVID-19-related hospitalization defined as admission up to 14 days after or 48 hours before a SARS-CoV-2 reinfection, and COVID-19-related death defined as death within 30 days of SARS-CoV-2 reinfection.

The potential confounders age, sex, comorbidity, country of origin, and staying at hospital were included in the analyses. Further, differences in test incidence between vaccinated and unvaccinated individuals, as well as changes in test incidence over time, e.g., due to changes in general test strategies may affect the results. Therefore, adjusting for test incidence was also included in the analyses. Information on age, sex, and country of origin was obtained from the CRS [11]. Comorbidity was defined as having a comorbidity diagnosis compatible with an increased risk of severe COVID-19 within 5 years prior to study entry. Diagnoses in the DNPR are coded according to the International Classification of Diseases, 10th revision (ICD-10) [14]. The ICD-10 codes used to define comorbidity diagnoses are shown in S3 Table. Definitions of country of origin is shown in S4 Table. Test incidence was the population test incidence rate, defined as number of persons having a test, divided by time in the respective risk periods, unvaccinated and periods after vaccination.

### Statistical analyses

Analyses were performed in three calendar periods with different dominant SARS-CoV-2 variants for all ages and for those 65 years of age or older. A variant period (Alpha, Delta, or Omicron) was defined as when a variant accounted for 75% or more of all whole genome sequenced PCR tests [17]. Lag periods between each period was introduced to avoid an overlap of variants. Individuals with a confirmed first-time SARS-CoV-2 infection were followed from 20 February 2021 until 15 June 2021 for the Alpha period, from 4 July to 20 November 2021 for the Delta period, and from 21 December 2021 to 31 January 2022 for the Omicron period. For all three variant periods, individuals were followed from start of follow-up or from 90 days after the date of the first infection, whichever came latest, and until death, immigration, receiving a booster vaccine, end of follow-up, or one of the following outcomes occurred, whichever came first: confirmed SARS-CoV-2 reinfection, COVID-19-related admission, or COVID-19-related death. Separate analyses were conducted for each variant period and each outcome.

We used a Poisson regression model accounting for overdispersion (quasi-Poisson) to estimate crude incidence rate ratios (IRRs), and a Cox proportional hazards regression model with underlying calendar time to estimate hazard ratios (HRs) adjusted for sex, age, country of origin, comorbidity (yes/no), staying at hospital, and test incidence before vaccination and in the respective time periods after vaccination. Sex, comorbidity, and country of origin were included as categorical variables, while age and staying at hospital were included as time-varying covariates. Test incidences were included as a numerical parameter.

The explanatory variable, vaccination, was included as a time-varying exposure, and individuals were considered completely vaccinated from 14 days or more after the last dose of a primary vaccination series, while a person was unvaccinated until receiving the first vaccine dose. The 14 days were used, as individuals receiving Spikevax, Vaxzevria, and Jcovden might not be fully protected before this time [18–20]. The time from receiving the first vaccine dose and until 13 days after receiving the second dose was excluded from the analyses. The proportional hazards assumptions were assessed graphically. VE was calculated as a percentage: *VE_crude_* = (1−*IRR*)∙100, and *VE_adjusted_* = (1−*HR*)∙100.

Data were analyzed using R version 4.1.2 (R Foundation for Statistical Computing; https://www.R-project.org/).

### Ethical considerations

In Denmark, approval from the Ethics Committee is not required for this type of study. The study adheres to the Strengthening the Reporting of Observational Studies in Epidemiology (STROBE) [21].

## Results

### Study population

The included populations for the variant periods comprised of 209,814 individuals with a SARS-CoV-2 infection before or during the Alpha period, 292,978 before or during the Delta period, and 245,530 before or during the Omicron period. Of these individuals, 40,281 (Alpha, 19.2%), 190,026 (Delta, 64.9%), and 158,563 (Omicron, 64.6%) completed a primary vaccination series during follow-up in the respective variant periods (Table 1). The mRNA vaccines, Comirnaty and Spikevax, accounted for more than 97% of the COVID-19 vaccines administered in all periods (Table 1). Individuals who completed a primary vaccination series in the Alpha period were older, predominantly female and more individuals had comorbidity when compared to the Delta and Omicron periods. Individuals completing a primary vaccination series in the Omicron period had the lowest median age (Table 1).

**Table 1:** Descriptive overview of the study population at start of follow-up.

	Alpha (B.1.1.7)		Delta (B.1.617.2)		Omicron (B.1.1.529)	
	20 February–15 June 2021		4 July–20 November 2021		21 December 2021–31 January 2022	
	Study population at entry (individuals with prior first-time SARS-CoV-2 infection)	Vaccinated individuals	Study population at entry (individuals with prior first-time SARS-CoV-2 infection)	Vaccinated individuals	Study population at entry (individuals with prior first-time SARS-CoV-2 infection)	Vaccinated individuals
**Number of individuals included, n (%)**	209,814 (100)	40,281 (19.2)	292,978 (100)	190,026 (64.9)	245,530 (100)	158,563 (64.6)
**Sex, n**						
Female (%)	106,717 (50.9)	24,200 (60.1)	146,111 (49.9)	95,623 (50.3)	120,432 (49.0)	77,595 (48.9)
Male	103,097	16,081	146,867	94,403	125,098	80,968
**Median age [IQR]**	35.3 [21.1–52.9]	63.5 [45.7–73.6]	32.0 [20.0–50.0]	41.2 [25.2–55.4]	25.4 [15.4–38.1]	27.6 [18.8–40.8]
**Vaccine product, n (%)**						
Comirnaty		34,383 (85.4)		161,115 (84.8)		134,046 (84.5)
Jcovden		1,057 (2.6)		1,629 (0.9)		466 (0.3)
Spikevax		4,781 (11.9)		27,201 (14.3)		24,020 (15.1)
Vaxzevria		60 (0.1)		81 (0.04)		31 (0.02)
**Country of origin, n (%)**						
Denmark	150,622 (71.8)	34,209 (85.0)	205,051 (70.0)	147,281 (77.5)	159,224 (64.8)	115,417 (72.8)
High-income	45,237 (21.6)	4,087 (10.1)	67,875 (23.2)	31,524 (16.6)	67,252 (27.4)	32,932 (20.8)
Other	13,670 (6.5)	1,976 (5.0)	19,896 (6.8)	11,182 (5.9)	18,963 (7.7)	10,179 (6.4)
Unknown	285 (0.1)	9 (0.02)	156 (0.05)	39 (0.02)	91 (0.04)	35 (0.2)
**Comorbidity, n (%)**	66,318 (31.6)	22,360 (55.5)	85,200 (29.1)	60,571 (31.9)	56,710 (23.1)	35,524 (22.4)

IQR, interquartile range; SARS-CoV-2, Severe Acute Respiratory Syndrome Coronavirus 2.

### SARS-CoV-2 reinfection

**Fig 1:**
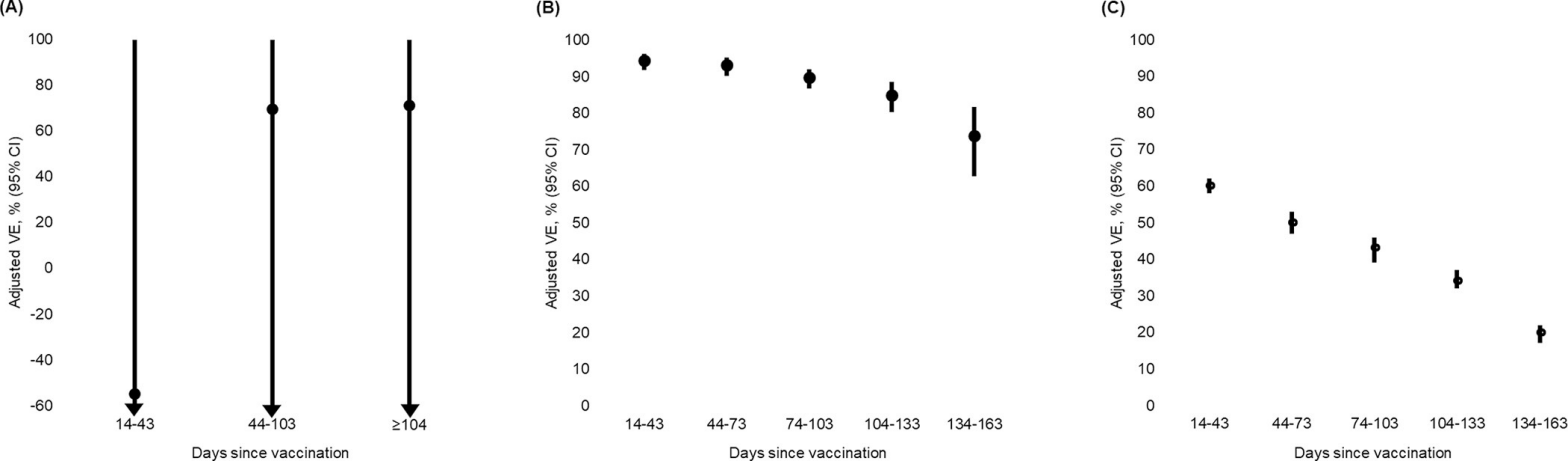
Adjusted VE against SARS-CoV-2 reinfection during periods of Alpha, Delta, or Omicron dominance. (**A**) Alpha variant (B.1.1.7), 20 February–15 June 2021. (**B**) Delta variant (B.1.617.2), 4 July–20 November 2021. (**C**) Omicron variant (B.1.1.529), 21 December 2021–31 January 2022. VE adjusted for age, sex, country of origin, comorbidity, staying at hospital, calendar time, and test incidence. CI, confidence interval; SARS-CoV-2, Severe Acute Respiratory Syndrome Coronavirus 2; VE, vaccine effectiveness.

During the Alpha period, 437 individuals had a confirmed SARS-CoV-2 reinfection (Table 2). The adjusted VE against reinfection was not statistically significant (−55%, 95% confidence interval (CI): -Inf to 100%) 14 to 43 days after vaccination, but rose to 69% (95% CI: -Inf to 100%) 44 to 103 days after vaccination and 71% (95% CI: -Inf to 100%) 104 days or more after vaccination (Fig 1, Table 2). During the Delta period, 1,678 individuals had a reinfection, resulting in an adjusted VE estimate against reinfection of 94% (95% CI: 92% to 96%) 14 to 43 days after vaccination. The VE declined from an initial 94% to 74% (95% CI: 63% to 82%) at 134 to 163 days after vaccination, after which it fluctuated due to too few events (Fig 1, Table 2). For reinfection during the Omicron period, the VE peaked at 60% (95% CI: 58% to 62%) 14 to 43 days after vaccination and declined to 20% (95% CI: 17% to 22%) 134 to 163 days after vaccination (Fig 1, Table 2). After this period, the VE fluctuated due to too few events. The VE decreased as time since vaccination increased during both the Delta and Omicron periods. Waning immunity following vaccination seemed to be more pronounced during the Omicron period.

**Table 2:** Adjusted VE against SARS-CoV-2 reinfection during periods of Alpha, Delta, or Omicron dominance.

	Alpha (B.1.1.7)							
	20 February–15 June 2021							
	Reinfections, n	PYRS	Adjusted VE against SARS-COV-2 reinfection*					
			VE		95% CI			
Previously infected and unvaccinated	405	41,435.95	1 (ref)		-			-
Previously infected and vaccinated (days since vaccination)								
14−43	22	2,053.82	−55%		-114,893%			100%
44−103	8	1,746.74	69%		−2,912%			100%
≥104	2	391.26	71%		−2,732%			100%
	**Delta (B.1.617.2)**							
	4 July–20 November 2021							
	Reinfections, n	PYRS		Adjusted VE against SARS-COV-2 reinfection*				
						95% CI		
				VE		2.5%	97.5%	
Previously infected and unvaccinated	1,373	33,375.63		1 (ref)		-	-	
Previously infected and vaccinated (Days since vaccination)								
14–43	26	12,249.40		94%		92%	96%	
44–73	30	12,887.70		93%		90%	95%	
74–103	65	11,693.72		90%		87%	92%	
104–133	64	7,399.62		85%		80%	88%	
134–163	43	3,712.30		74%		63%	82%	
164–193	23	2,231.93		80%		68%	88%	
194–223	15	1,027.79		74%		55%	85%	
224–253	5	473.47		76%		40%	90%	
254–283	3	207.27		81%		39%	94%	
≥284	2	23.37		42%		−143%	86%	
	**Omicron (B.1.1.529)**							
	21 December 2021–31 January 2022							
	Reinfections, n	PYRS		Adjusted VE against SARS-COV-2 reinfection*				
						95% CI		
				VE		2.5%	97.5%	
Previously infected and unvaccinated	24,002	7,712.27		1 (ref)		-	-	
Previously infected and vaccinated (days since vaccination)								
14–43	2,033	1,690.69		60%		58%	62%	
44–73	1,271	746.05		50%		47%	53%	
74–103	1,061	702.72		43%		39%	46%	
104–133	3,014	1,954.08		34%		32%	37%	
134–163	6,737	3,271.95		20%		17%	22%	
164–193	3,195	1,189.79		14%		10%	17%	
194–223	502	209.88		21%		13%	28%	
224–253	161	71.18		22%		8%	35%	
254–283	53	32.57		41%		22%	55%	
284–313	32	17.06		29%		−1%	50%	
314–343	25	13.09		28%		−8%	52%	
≥344	20	6.14		31%		−8%	56%	

CI, confidence interval; PYRS, person-years; SARS-CoV-2, severe acute respiratory syndrome coronavirus 2; VE, vaccine effectiveness.

*Adjusted for age, sex, country of origin, comorbidity, staying at hospital, calendar time, and test incidence.

VE against hospitalization and death were also analyzed, but due to too few events, it was not possible to estimate a VE. During the Alpha period, 32 COVID-19-related hospitalizations were recorded in unvaccinated individuals (incidence rate (IR): 0.001), while 8 were recorded among those who had been vaccinated (IR: 0.002). During the Delta period, 13 COVID-19-related hospitalizations were recorded among unvaccinated individuals (IR: 0.0004) and 10 among vaccinated individuals (IR: 0.0002). For the Omicron period, these numbers were 61 (IR: 0.007) and 37 (IR: 0.004), respectively.

For COVID-19-related death, only 8 events were recorded during the three periods: 3 during the Alpha period, 3 during the Delta period, and 2 during the Omicron period. Therefore, it was not possible to report any results for this outcome.

Due to the vaccine rollout prioritizing the elderly, some age difference is seen between the groups in Table 1. To compare the VE in a comparable age group, we restricted the analyses to those 65 years of age or older. Again, there was too few events to estimate VE. The proportion of events among the elderly was lowest for reinfections at a maximum of 11% during the Alpha period, while for severe outcomes, up to 48% of hospitalizations and 100% of deaths occurred in this age group (S2 Table). The proportion of events in the elderly was markedly lower for reinfections and COVID-19-related hospitalizations during the Omicron period than for the Alpha and Delta periods (S2 Table). The proportional hazards assumptions were found to be valid for all analyses.

## Discussion

In this nationwide, population-based cohort study, we found a primary COVID-19 vaccination series to be associated with a significant VE against SARS-CoV-2 reinfection during periods dominated by the Alpha, Delta, or Omicron variants.

For the Delta and Omicron periods, the VE against reinfection was highest 14 to 43 days after completed primary vaccination series, although generally lower against the Omicron variant. In the Alpha-dominated period, the VE was not statistically significant with 95% CI’s including zero. During the Alpha period, the oldest and most vulnerable individuals were vaccinated, including those with a less responsive adaptive immune system and antibody production [22–24]. A slower immune response following vaccination might explain why the VE was not statistically significant. A slower clearance of previous infections has been observed in the elderly Danish population [6]. Thus, a minimum of 90 days between two positive tests might not be sufficient to clear a SARS-CoV-2 infection in the elderly, which would lead to an underestimated VE in this population. In addition, a Danish cohort study showed that natural protection against reinfection during the Alpha period was 83.4% (95% CI: 82.2 to 84.6%), while it decreased in the Delta and Omicron periods if the initial infection was more than 1 year ago [8]. That is, during the Alpha period, reinfections might have been less likely due to a higher natural protection gained from recent, initial infections. In the Delta period, a VE of 94% (95% CI: 92% to 96%) against reinfection was observed 14 to 43 days after vaccination. This is in accordance with observational studies from Sweden and Israel, in periods of both Alpha and Delta [25] or Delta dominance [2] where the VE ranged from 66% (95% CI: 61% to 69%) [25] to 82% (95% CI: 80% to 84%) [2]. A prospective cohort study from the United Kingdom reported similar levels of hybrid protection against reinfection, lasting for more than a year [26]. The longevity of protection seen in the UK study differs from our result, where the VE during the Delta period decreased from the initial 94% to 74% (95% CI: 63% to 82%), 134 to 163 days postvaccination.

For the Omicron period, our study showed an initial VE against reinfection of 60% (95% CI: 58% to 62%), which is lower than what we found for the other variants, but still indicates an additional protection following vaccination. This is similar to a study from Qatar, where a VE of 55.1% (95% CI: 50.9% to 58.9%) against reinfection with the Omicron variant after two doses of Comirnaty was estimated [27]. A lower VE of two doses COVID-19 mRNA vaccines against hospitalization was also seen in a period dominated by Omicron (34.6%, 95% CI: 25.5% to 42.5%) compared to Delta (47.5%, 95% CI: 38.8% to 54.9%), in a test-negative design study from the USA [28].

Regarding COVID-19-related admission during the Alpha period, a seemingly higher risk was observed for vaccinated individuals (IR: 0.002) compared to those without vaccination (IR: 0.001). Here, the same issue as for the VE against reinfection in this period might be at play. More so, the elderly and most vulnerable or at-risk individuals were prioritized for vaccination during a period of high incidence of SARS-CoV-2 and with outbreaks at several long-term care facilities (LTCFs). Therefore, it was somewhat expected that the risk of severe outcomes is more pronounced in the vaccinated compared to the unvaccinated in the Alpha period. We also cannot rule out that some LTCF residents and healthcare workers were infected at the time of vaccination, or shortly after. This increases the risk of SARS-CoV-2 infections being identified after vaccination, even though the infection happened before. Similar observations were reported in the International Severe Acute Respiratory Infection Consortium Clinical Characterisation Protocol from the UK [29].

In the present study, it was not possible to estimate VE for COVID-19-related hospitalization or death. This was partly due to too few events, but might also stem from a broad definition of these outcomes, resulting in some uncertainty regarding whether or not a SARS-CoV-2 infection was the main reason for hospitalization or death. However, a broad definition allows for inclusion of patients who are hospitalized or dead due to COVID-19 but have not been classified as such using ICD-10 codes. A goal for future studies will be to ascertain the level of completeness for COVID-19-specific ICD-10 codes.

Among those 65 years or older, we saw a markedly increased proportion of cases for severe outcomes compared to SARS-CoV-2 reinfections in the same variant period and overall (S2 Table). A meta-analysis comparing mortality in 5 different countries also found age to be a major factor in increased mortality [30]. However, despite a follow-up time of approximately 9 months, the recorded number of severe outcomes is very low, indicating that previous infection and vaccination offer protection against severe disease, also in this older age group.

Both mRNA and non-mRNA vaccines were included to increase the statistical strength of the study. Since non-mRNA vaccines constitute less than 3% of administered vaccines, it is not likely that any differences in VE between the vaccine types influence the results.

A major strength of this study is the completeness of the Danish registries, which reduces the risk of selection bias as they cover all individuals residing in Denmark and their contacts with vaccination or test centers, as well as the Danish personal registration number ensuring individual-level linkage of data. Also, Denmark has had one of the highest testing rates in the world [31], which limits the risk of undiscovered reinfections.

This study also has some limitations. Despite the high test rate, we cannot rule out undetected reinfections, especially asymptomatic infections among vaccinated individuals, which might inflate the VE. We also cannot rule out that vaccinated and unvaccinated individuals had different health-seeking behavior or risk behavior, which could affect VE.

In summary, this study showed that among previously infected individuals who have completed a primary vaccination series, there is a significant VE against SARS-CoV-2 reinfection for the SARS-CoV-2 variants Alpha, Delta, and Omicron, lasting up to 9 months. Even though vaccination seems to protect to a lesser degree against reinfection with the Omicron variant, these findings are of public health relevance as they show that previously infected individuals still benefit from COVID-19 vaccination in all three variant periods.

## Supporting information

S1 STROBE Statement Checklist of items that should be included in reports of *cohort studies*. (DOC)

S1 Table Crude vaccine effectiveness against SARS-CoV-2 reinfection during periods of Alpha, Delta, or Omicron dominance. (DOCX)

S2 Table Events in the study population (all ages) and proportion among those 65 years or older during periods of Alpha, Delta, or Omicron dominance. (DOCX)

S3 Table Overview of ICD-10 codes included in the comorbidity variable. (DOCX)

S4 Table Definition of country of origin. (DOCX)

## Abbreviations

CI: confidence interval
COVID-19: Coronavirus Disease 2019
CRS: Civil Registration System
DNPR: Danish National Patient Registry
DVR: Danish Vaccination Registry
HR: hazard ratio
ICD-10: International Classification of Diseases, 10th revision
IR: incidence rate
IRR: incidence rate ratio
LTCF: long-term care facility
RT-PCR: reverse transcription polymerase chain reaction
SARS-CoV-2: Severe Acute Respiratory Syndrome Coronavirus 2
VE: vaccine effectiveness
