# Supplementary material for: Vaccine effectiveness against SARS-CoV-2 reinfection during periods of Alpha, Delta, or Omicron dominance: A Danish nationwide study
Source: PLoS Med. 2022 Nov 22;19(11):e1004037. doi: 10.1371/journal.pmed.1004037 (PMC9681105; doi:10.1371/journal.pmed.1004037)
Supplement: S1 Table — (DOCX) [file pmed.1004037.s002.docx]

## Supplementary

##### Table S1. Crude vaccine effectiveness against SARS-CoV-2 reinfection during periods of Alpha, Delta or Omicron dominance

|  | **Alpha (B.1.1.7)** | | | | |
| --- | --- | --- | --- | --- | --- |
|  | 20 February - 15 June 2021 | | | | |
|  | Reinfections, n | PYRS | Crude vaccine effectiveness against  SARS-COV-2 reinfection | | |
|  |  |  | VE | 95% CI | |
| Previously infected and unvaccinated | 405 | 41,435.95 | 1 (ref) | - | - |
| Previously infected and vaccinated (Days since vaccination) |  |  |  |  |  |
| 14-43 | 22 | 2,053.82 | -10% | -180% | 57% |
| 44-103 | 8 | 1,746.74 | 53% | -116% | 90% |
| ≥104 | 2 | 391.26 | 48% | -985% | 97% |

Abbreviations: CI, confidence interval; PYRS, person-years; SARS-CoV-2, severe acute respiratory syndrome coronavirus 2; VE, vaccine effectiveness

|  | **Delta (B.1.617.2)** | | | | |
| --- | --- | --- | --- | --- | --- |
|  | 4 July - 20 November 2021 | | | | |
|  | Reinfections, n | PYRS | Crude vaccine effectiveness against  SARS-COV-2 reinfection | | |
|  |  |  |  | 95% CI | |
|  |  |  | VE | 2.5% | 97.5% |
| Previously infected and unvaccinated | 1,373 | 33,375.63 | 1 (ref) | - | - |
| Previously infected and vaccinated (Days since vaccination) |  |  |  |  |  |
| 14-43 | 26 | 12,249.40 | 95% | 90% | 97% |
| 44-73 | 30 | 12,887.70 | 94% | 89% | 97% |
| 74-103 | 65 | 11,693.72 | 86% | 79% | 91% |
| 104-133 | 64 | 7,399.62 | 79% | 67% | 87% |
| 134-163 | 43 | 3,712.30 | 72% | 51% | 84% |
| 164-193 | 23 | 2,231.93 | 75% | 47% | 88% |
| 194-223 | 15 | 1,027.79 | 65% | 10% | 86% |
| 224-253 | 5 | 473.47 | 74% | -28% | 95% |
| 254-283 | 3 | 207.27 | 65% | -178% | 96% |
| ≥284 | 2 | 23.37 | -108% | -2516% | 83% |

Abbreviations: CI, confidence interval; PYRS, person-years; SARS-CoV-2, severe acute respiratory syndrome coronavirus 2; VE, vaccine effectiveness

##### Table S1. Continued

|  | **Omicron (B.1.1.529)** | | | | |
| --- | --- | --- | --- | --- | --- |
|  | 21 December 2021 - 31 January 2022 | | | | |
|  | Reinfections, n | PYRS | Crude vaccine effectiveness against  SARS-COV-2 reinfection | | |
|  |  |  |  | 95% CI | |
|  |  |  | VE | 2.5% | 97.5% |
| Previously infected and unvaccinated | 24,002 | 7,712.27 | 1 (ref) | - | - |
| Previously infected and vaccinated (Days since vaccination) |  |  |  |  |  |
| 14-43 | 2,033 | 1,690.69 | 61% | 59% | 64% |
| 44-73 | 1,271 | 746.05 | 45% | 41% | 50% |
| 74-103 | 1,061 | 702.72 | 51% | 47% | 56% |
| 104-133 | 3,014 | 1,954.08 | 50% | 48% | 53% |
| 134-163 | 6,737 | 3,271.95 | 34% | 31% | 36% |
| 164-193 | 3,195 | 1,189.79 | 14% | 9% | 18% |
| 194-223 | 502 | 209.88 | 23% | 12% | 32% |
| 224-253 | 161 | 71.18 | 27% | 9% | 42% |
| 254-283 | 53 | 32.57 | 48% | 22% | 65% |
| 284-313 | 32 | 17.06 | 40% | 0% | 64% |
| 314-343 | 25 | 13.09 | 39% | -9% | 65% |
| ≥344 | 20 | 6.14 | -5% | -99% | 45% |

Abbreviations: CI, confidence interval; PYRS, person-years; SARS-CoV-2, severe acute respiratory syndrome coronavirus 2; VE, vaccine effectiveness
