# Supplementary material for: Vaccine effectiveness against SARS-CoV-2 reinfection during periods of Alpha, Delta, or Omicron dominance: A Danish nationwide study
Source: PLoS Med. 2022 Nov 22;19(11):e1004037. doi: 10.1371/journal.pmed.1004037 (PMC9681105; doi:10.1371/journal.pmed.1004037)
Supplement: S2 Table — (DOCX) [file pmed.1004037.s003.docx]

## Supplementary

##### Table S2. Events in the study population (all ages) and proportion among those 65 years or older during periods of Alpha, Delta or Omicron dominance

|  | **Events in the study population and among those ≥65 years old** | |
| --- | --- | --- |
| Outcome and variant period | Total no. of events | Events in those ≥65 years old (%) |
| **SARS-CoV-2 reinfection** |  |  |
| Alpha | 437 | 48 (11) |
| Delta | 1,678 | 34 (2) |
| Omicron | 42,106 | 203 (0.5) |
| Overall | 44,221 | 285 (0.6) |
| **COVID-19 related hospitalization** |  |  |
| Alpha | 40 | 19 (48) |
| Delta | 23 | 9 (39) |
| Omicron | 98 | 4 (4) |
| Overall | 161 | 32 (20) |
| **COVID-19 related death** |  |  |
| Alpha | 3 | 3 (100) |
| Delta | 3 | 2 (67) |
| Omicron | 2 | 2 (100) |
| Overall | 8 | 7 (88) |
