# Supplementary material for: Vaccine effectiveness against SARS-CoV-2 reinfection during periods of Alpha, Delta, or Omicron dominance: A Danish nationwide study
Source: PLoS Med. 2022 Nov 22;19(11):e1004037. doi: 10.1371/journal.pmed.1004037 (PMC9681105; doi:10.1371/journal.pmed.1004037)
Supplement: S4 Table — (DOCX) [file pmed.1004037.s005.docx]

## Supplementary

##### Table S4. Definition of country of origin

| **Country of origin** | **Definition** |
| --- | --- |
| Danish | Individuals who were born in Denmark or abroad and have at least one parent who is a Danish citizen and born in Denmark. |
| High-income | Individuals with country of origin*: nordic countries, EU countries, Andorra, Liechtenstein, Monaco, San Marino, Switzerland, United Kingdom, the Vatican City, Canada, USA, Australia or New Zealand. |
| Other | Individuals with country of origin*: all other countries than the countries defined as high-income. |

*Country of origin is defined as follows:

- When neither parent is known, the country of origin is defined on the basis of the person's own information. If the person is an immigrant, it is assumed that the country of origin is equal to the country of birth. If the person is a descendant, it is assumed that the country of origin is equal to the country of citizenship.
- When only one parent is known, the country of origin is defined based on that parent’s country of birth. If this is Denmark, the country of citizenship is used.
- When both parents are known, the country of origin is defined on the basis of the mother's country of birth and country of citizenship, respectively.
